# Longitudinal Case Study of Regression-Based Hand Prosthesis Control in Daily Life

**DOI:** 10.3389/fnins.2020.00600

**Published:** 2020-06-17

**Authors:** Janne M. Hahne, Meike A. Wilke, Mario Koppe, Dario Farina, Arndt F. Schilling

**Affiliations:** ^1^Applied Rehabilitation Technology Lab, Department of Trauma Surgery, Orthopedic Surgery and Hand Surgery, University Medical Center Göttingen, Göttingen, Germany; ^2^Faculty of Life Sciences, University of Applied Sciences (HAW) Hamburg, Hamburg, Germany; ^3^Global Research and Innovation Hub, Ottobock SE & Co. KGaA, Duderstadt, Germany; ^4^Department of Bioengineering, Imperial College London, London, United Kingdom

**Keywords:** Myolectric control, prosthesis, regression, simultaneous control, clinical evaluation

## Abstract

Hand prostheses are usually controlled by electromyographic (EMG) signals from the remnant muscles of the residual limb. Most prostheses used today are controlled with very simple techniques using only two EMG electrodes that allow to control a single prosthetic function at a time only. Recently, modern prosthesis controllers based on EMG classification, have become clinically available, which allow to directly access more functions, but still in a sequential manner only. We have recently shown in laboratory tests that a regression-based mapping from EMG signals into prosthetic control commands allows for a simultaneous activation of two functions and an independent control of their velocities with high reliability. Here we aimed to study how such regression-based control performs in daily life in a two-month case study. The performance is evaluated in functional tests and with a questionnaire at the beginning and the end of this phase and compared with the participant’s own prosthesis, controlled with a classical approach. Already 1 day after training of the regression model, the participant with transradial amputation outperformed the performance achieved with his own Michelangelo hand in two out of three functional metrics. No retraining of the model was required during the entire study duration. During the use of the system at home, the performance improved further and outperformed the conventional control in all three metrics. This study demonstrates that the high fidelity of linear regression-based prosthesis control is not restricted to a laboratory environment, but can be transferred to daily use.

## Introduction

Losing a hand has a dramatic impact to a person’s life. Myoelectric hand prostheses can reduce the repercussions and help the person to conduct activities of daily living with less restrictions. Conventionally, two electrodes placed on antagonistic muscles are used to control a single degree of freedom (DOF) of the hand (Muzumdar, 2004), i.e., opening and closing the hand. Mode-switching techniques, such as co-contraction are used control a second DOF such as a wrist rotation or other functions, such as different grip types sequentially, which is cumbersome and limits the benefit of additional functions (Amsuess et al., 2014).

To overcome the limitations, classification techniques (Englehart and Hudgins, 2003; Oskoei and Hu, 2007; Peerdeman et al., 2011; Scheme and Englehart, 2011; Hahne et al., 2012) have been applied that compare the current electromyographic (EMG) with training-patterns with known motion. The classifier decides for the most similar class, allowing for directly accessing all functions, although typically only in a sequential manner. Recently, classification based control approaches have become clinically available (Coapt-LLC, 2019; Ottobock, 2019).

In the past years also regression algorithms have been applied in prosthetic research (Jiang et al., 2009; Ameri et al., 2014; Gijsberts et al., 2014; Hahne et al., 2014). The fundamental difference to classification is, that a regressor does not decide for a particular motion class. Instead, a regressor estimates activity levels for all DOFs simultaneously. This allows not only performing two different functions at the same time but even to control their velocity independently. Since the output reacts to any changes of the EMG input, the user can more easily compensate for disturbances, which increases the reliability (Hahne et al., 2017).

The relatively high classification/regression performance shown in laboratory conditions may not necessarily translate into good functional recovery in real prosthetic use (Jiang et al., 2012). Factors such as changes in arm position (Fougner et al., 2011; Khushaba et al., 2016; Beaulieu et al., 2017), small electrode displacements (Young et al., 2011; Hwang et al., 2017), sweat, mechanical load to the socket (Cipriani et al., 2011), or time between training and application of the algorithm (Amsuss et al., 2013; Vidovic et al., 2016) can degrade the performance and lead to an unreliable control in daily life.

Recently, we have shown a relatively high robustness of the regression approach in five prosthetic users during advanced clinical tests in the laboratory that involved challenging arm positions and the application on a second day without retraining (Hahne et al., 2018). The purpose of this eight-week case study was to test a research prosthesis controlled by linear regression (LR) under fully uncontrolled conditions in the daily life and compare it with the participant’s own prosthesis with a conventional control (CC).

## Methods

### Participant

**FIGURE 1:**
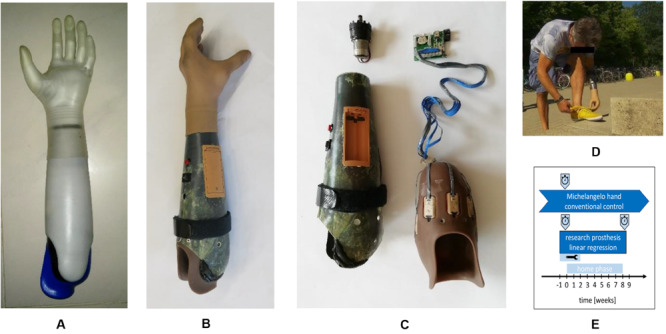
Prostheses hardware used in this study. **(A)** Michelangelo hand owned by the participant and used as a baseline with conventional two-channel control in this study. **(B)** Research prosthesis controlled by eight channels and linear regression. **(C)** Components of the research prosthesis: rotation unit (upper left), outer socket with battery holder, power-switch and strap with hook and loop fastener to adjust the fit (lower left), inner socket made from silicone with eight integrated electrode modules (lower right), customized controller. **(D)** Use of the regression based prosthesis in uncontrolled conditions, in daily life. **(E)** Chronology of this case report indicating prosthetic use, functional assessments (stopwatch), and adjustments period (tool icon). Michelangelo hand was used already since 12 month at the study period and before the participant used single-DOF prostheses for around 35 years.

The participant of this case study was a 58-year old man, who got his left hand amputated on trasradial level, 35 years before this study. Since that time he has been actively using conventional myoelectric prostheses controlled with two EMG channels. Until approximately 12 months before the beginning of this study he was wearing only single-DOF prostheses without rotation. Then he was provided with an Otto Bock Michelangelo hand and used conventional slope-control to access grasp and rotation and co-contraction to alter between the two grip functions. He had moderate experience with both classification- and regression-based control approaches from earlier experiments and was familiar with the functional tests conducted in this study. Due to his participation in our previous laboratory study (Hahne et al., 2018) with a similar system, he was already familiar with the control concept and was able to generate suitable training data. A chronologic overview on the participant’s prosthetic history and this study is provided in Figure 1E.

### Prosthesis

The research prosthesis used in this study was an Otto Bock VariPlus Speed hand with electric wrist rotator. A customized socket was built for the participant (inner socket high temperature vulcanization silicone incl. eight Otto Bock 13E200 electrodes, outer socket laminated carbon fiber). It included a customized controller, a battery pack and an easily accessible power-switch and allowed for simultaneous and proportional control of the two DOFs with LR.

The system and the training procedure were similar to those described in Hahne et al. (2018). First, four suitable phantom-limb motions were selected based on visual inspection of the EMG (phantom flexion/extension for closing/opening, pronation/little-finger flexion for rotation). The latter gave a relatively strong and clear pattern and was chosen instead supination to increase the robustness.

For the algorithmic training, data with known movement association was recorded. Therefore, the participant was asked to follow trapezoidal contraction profiles for all four motions (2 s rest, 3 s ramp-up, 3 s static contraction, and 2 s ramp-down). The entire training dataset consisted of only one repetition of each motion in neutral arm position, corresponding to a total of 40 s of data for training the algorithm (data in Supplementary Material).

A linear mapping model *W* from the eight-dimensional EMG envelopes *x* to the two-dimensional control signal $\hat{y}$ (Eq. 1) was established by ordinary LR (Eq. 2), where *X* and *Y* are matrices with the collected training data and labels based on the visual cues:

(1) $$\hat{y}=W^{T}\,x$$

(2) $$W={\left({\mathrm{XX}}^{T}\right)}^{-1}\,{\mathrm{XY}}^{T}$$

Algorithmic training of the regression model was conducted with a customized MATLAB framework on a standard PC (I7, 2 × 2.5 GHz, 16 GB RAM, and Windows 7). As previously shown (Hahne et al., 2014), a linear regressor on EMG can estimate simultaneous activations of two DOFs with a clinically feasible number of electrodes, even when trained on non-combined motions only. Following the training of the algorithm, a real-time control of a cursor in a two-dimensional coordinate system was established in position-control mode to verify proper control.

As in CC, the prosthesis was operated in velocity-control mode. The stronger the participant contracted, the faster the prosthesis moved and at relaxation, the prosthesis did not move back. The envelope output of the active EMG electrodes could be directly utilized without windowing or feature extraction.

To suppress unintended motions and fine-tune the velocity of the prosthesis, two thresholds were individually adjusted for each of the four prosthetic functions that determined the level of activation and the level for that the maximal speed is reached. Additionally, the customized controller contained a real-time clock and a micro-SD card that was used for continuous recording of the EMG envelopes to allow for quantitative usage evaluation. For the analysis, we considered only reconstructed motions with speed larger than five percent of the maximal speed and a duration larger than 200 ms. A motion that included a phase with both DOFs active was counted as one multi-DOF motion. The results of this analysis were averaged over periods of 1 week of the home phase.

As a baseline, we compared our research prosthesis (controlled with LR) with the Michelangelo hand owned by the participant and used daily before this study for approximately 12 months. It was controlled by two EMG channels on the residual flexor and extensor muscles and a CC technique based on the initial EMG slope (Muzumdar, 2004). Slowly increasing EMG amplitudes would open/close the prosthesis while quickly raising contractions would rotate the hand with a velocity proportional to the EMG amplitude and a co-contraction was used to change between lateral and palmar grip.

### Functional and Subjective Assessment

The functional performance of the LR-controlled research-prototype prosthesis was assessed with three standardized tests during laboratory sessions performed at the beginning of the study, 1 day after the training with the new system, and at the end of the 2-month home phase. The CC-controlled Michelangelo hand was evaluated with the same functional tests at the beginning of the study only. Since the participant had already used this fitting for 12 months in daily life, we assumed that the training with this prosthesis was finished and the performance already saturated. The functional tests performed were the Box-And-Blocks Test (Mathiowetz et al., 1985), the Clothespin-Relocation Test (Hussaini and Kyberd, 2017), and the Southampton Hand Assessment Procedure (SHAP; Kyberd et al., 2009).

The Box-And-Blocks Test requires to transfer as many wooden blocks as possible from one box into another within 60 s. The Clothespin-Relocation Test assesses the time needed to relocate three pins (10 N grip force) of the Rolyan Graded Pinch Exerciser from a horizontal to a vertical bar. For the SHAP test times for a broad spectrum of activities of daily living are measured and compared with a normative database of young healthy people (Light et al., 2002). A SHAP-score of 100 corresponds to normal and 0 to minimal functionality.

The Box-And-Blocks Test and the Clothespin-Relocation Test were performed ten times in each laboratory session, in order to reduce the scatter and test for statistically significant differences within the participant. Statistical comparisons were performed with a Wilcoxon rank-sum test with Bonferroni correction and a threshold of *p* = 0.05.

Beside the functional tests in the laboratory, we aimed to gather information regarding the reliability in other daily life situations, where disturbing factors that were not present in the laboratory tests appear. Also, we were interested in the personal opinion of the participant regarding the new control approach. Therefore, he was asked to fill in customized questionnaires for the first and the last week of the home phase (one pencil paper form covering both prostheses, not-validated). He had to grade different aspects of the research prosthesis and his own Michelangelo hand on a scale from 0 to 10 (questions in Supplementary Material).

The study was conducted in accordance with the declaration of Helsinki and was approved by the local ethic commission (approval number 23/4/16) and written, informed consent was obtained.

## Results

### Summary of Home Phase

During the home phase of this study the participant was motivated to use the LR-controlled research prosthesis as much as possible, but he was allowed to use his own Michelangelo hand. His previous single-DOF prosthesis was not used and he reported to wear a prosthesis most of the time.

In the first two weeks of the home phase, problems with the socket fitting including electrode lift offs required several iterations of corrections (Figure 1E). Therefore, he visited our laboratory several times and was visited by a technician once in that time. Adding a strap adjuster system to the outer socket finally allowed the subject to control the tightness of the socket to ensure proper fixation and comfortable fit and to compensate for stump volume variations.

No retraining of the regression model that transforms the eight EMG-envelopes into control signals was required during the entire study. Only the thresholds that were used as a post-processing after the regression step to fine-tune the speed and to reduce the risk of unintended activations were adjusted as the participant experienced the sensitivity of the control as too high in the first week at home. On day 13 all thresholds were therefore increased by 75%, followed by corrections for the upper thresholds for hand open and supination. These were the only adjustments made on the controller during the home phase. After these mechanical and parametrical adjustments, the participant reported to be very satisfied with the control for the rest of the study. No further laboratory visits were required during the home phase but we called him occasionally to verify that everything was ok. He used the LR prosthesis in any activity of daily life, such as cooking, eating, cleaning, dressing, or fastening his shoes (Figure 1). He further reported that the LR control was very intuitive and that he could easily change from CC to LR. In contrast, when changing back to CC, he always needed some time of familiarization to the slope-control. He reported unintended rotations with his Michelangelo hand, especially in situations when he was in a rush and therefore generated quickly rising EMG amplitudes; a problem that was already present before this study. Being unsatisfied with his CC, he requested that the rotation function to be removed from his Michelangelo prosthesis by his prosthetist during the time of this study. We did not modify the control of the participant’s Michelangelo hand and no other changes beside removing the rotation were made externally during this study.

Despite satisfaction with the control, he, however, did not constantly wear the research prosthesis. He explained this choice with a preferred esthetic appearance of his Michelangelo hand over the VariPlus Speed hand and a more comfortable socket. He did not report any injuries, blisters, muscle aches, headaches, or similar issues related to the prostheses during the study. Overall, he indicated that he would prefer the LR control algorithm to be embedded in his own Michelangelo prosthesis and socket.

### Data Log

**FIGURE 2:**
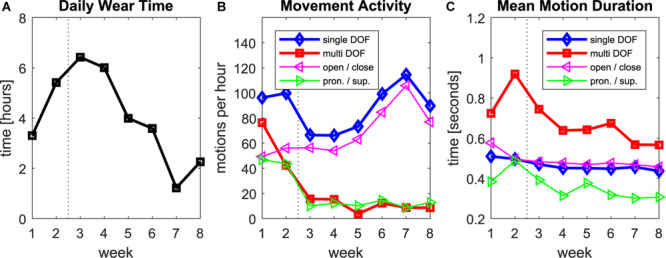
Data log of the regression prosthesis during the home phase of the study. **(A)** Daily wear time, average per week. **(B)** Counts of single and multi-DOF motions per hour of wear time. **(C)** Average duration of each individual motion. In all plots, the dashed vertical line indicates the time, when final adjustments to the socket and the parameters were finalized.

The average amount of time the participant was wearing the research prosthesis increased within the first weeks and remained on a relatively high level between five and seven hours per day until week four (Figure 2A). In the second half of the study the average wear time decreased to 1–4 h per day. The wear-time of his own Michelangelo hand was not tracked as this was not possible with the commercial hardware, however, one can assume that it behalves complementary to the research prothesis, as the participant stated to wear a prothesis all day long. The number of motions he conducted per hour while wearing the research prosthesis was relatively high at the beginning of the study, with approximately 100 single-DOF motions and 80 multi-DOF motions, and decreased after the final adjustments in the end of week 2 to approximately 70 single-DOF and less than 20 multi-DOF motions per hour (Figure 2B). The amount of single DOF motions increased in the second half of the study to reach 120 motions per hour toward the end of the study. The multi-DOF motions on the other hand remained low in number until the end of the study. When considering the frequency of single DOF motions for each DOF separately, both DOFs were almost equally often active in the first two weeks. After week 2, however, the use of rotation decreased to approximately 10 motions per hour and remained at this level until the end of the study. The number of motions in the open/close DOF did not change in the first weeks and increased in the second half of the study from less than 60 to 80–100 motions per hour. The duration of single-DOF motions was shorter than multi-DOF motions and rotations wereshorter than motions of the DOF hand open/close (Figure 2C). There is a small trend towards decreasing duration for all motions over the time of the study.

### Functional Tests

**FIGURE 3:**
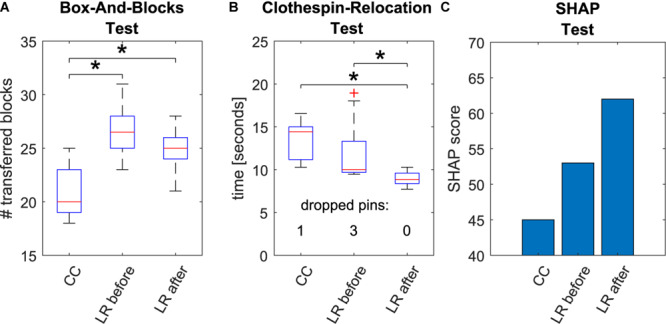
Results of the functional tests. Box-And-Blocks Test **(A)**, Clothespin-Relocation Test **(B)**, and SHAP Test **(C)**. All tests were conducted with the conventionally controlled Michelangelo hand owned by the participant (CC) and the regression-based research prosthesis (LR before) in the beginning of the study. The regression control was evaluated a second time after the 8-week home phase (LR after). For Box-And-Blocks and Clothespin-Relocation Test 10 repetitions were conducted each time to apply intra-subject statistics. Statistically significant differences (*p* < 0.05) are marked with asterisks.

All three functional tests were performed with the participant’s own Michelangelo hand (in beginning only) and the research prosthesis (before and after home phase; Figure 3).

In the Box-And-Blocks Test the participant performed significantly better with LR prototype than with CC (*p* < 0.05) already before the home phase. The performance in this test did not further improve after the home phase but remained significantly better than CC in the second evaluation. Performance of the Clothespin-Relocation Test with the LR control was not significantly different with respect to CC before the home phase. However, after the home phase, the control with the regression-based control (LR after) improved significantly compared to both methods prior to training. Also, while in the initial session, one and three pins were dropped in total with CC and LR, respectively, after the home phase no pin was dropped with LR. In the SHAP Test the participant reached higher SHAP score with the regression (score 53) with respect to CC (score 45) already at the beginning of the study and further improved after the home phase (score 62).

### Subjective Assessment

**FIGURE 4:**
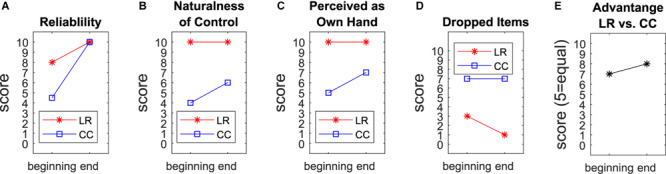
Questionnaires. The participant graded for the first and last week of the home phase the reliability **(A)**, naturalness of control **(B)**, to which extent he perceived the prosthesis as his own hand **(C)**, and the frequency of dropped or unintendedly released items **(D)** for both prostheses. In these metrics, LR scored better than CC at each time point. He reported a moderate advantage of LR compared to CC **(E)**.

At the beginning of the study, the participant graded the reliability of the regression control higher than the one of his own prosthesis (Figure 4A). In the end both prostheses got full scores. This evaluation followed the longitudinal experience and final adjustments of the socket and the threshold parameters in LR and the deactivation of the rotation function for the Michelangelo hand. The naturalness of the control and the perception of the prosthesis as own hand were rated with maximal scores in the beginning and the end for LR while CC got only moderate scores, slightly increasing in the end (Figures 4B,C). The frequency of dropped items during the home phase was rated higher for CC. This score further improved with LR at the end of the study (Figure 4D). The participant graded a moderate advantage of LR in comparison with CC, slightly increasing at the end (Figure 4E).

## Discussion

We evaluated a regression-based controller for simultaneous and proportional control of a 2-DOF prosthetic hand for 8 weeks in daily use. The regression model was trained with data recorded in less than 1 min and no retraining was required during the 2 months. In the first two weeks we encountered some reliability issues (mainly unintended rotations), that were fixed by modifications on the socket and final adjustments of the thresholds. The participant was then very satisfied with the control and did not report any further reliability issues. The strong decrease of rotation and multi-DOF motions after the adjustments in the second week (Figure 2B) could be an indication that many of these motions during the first two weeks were unintended activations.

As expected, the motion counts after week two revealed that the hand DOF (open/close) was more important than the wrist rotation for the participant. Hand use occurred 5 to 10 times more often than wrist rotation. Nevertheless, the participant reported to find the rotation useful, especially due to the simultaneous and intuitive control. The control of his own prostheses was in contrast perceived as so unphysiological and slow that he decided to remove the rotation there.

Multi-DOF motions were as frequent as rotations. This could be an indication for a physiological use of the hand, where in preparation of a grasp rotation is, e.g., combined with opening of the hand. In this light, the longer duration of multi-DOF motions could be explained by including the whole preparation movement in one Multi-DOF motion. Such more natural motions are not possible with current commercial control systems, where the user has to activate the individual functions sequentially. After the adjustment of the thresholds the participant reported that he did not notice any false activations of prosthesis functions in daily life. However, there is no final proof that all recorded multi-DOF motions were intended by the participant of the study.

The average daily wear time of the prosthesis decreased toward the end of the study, which could be a sign of dissatisfaction with the control. However, at the same time, the number of grasp motions per hour increased. Together, this could indicate that the participant used the prosthesis especially for physically active tasks and changed to his Michelangelo for less active phases, as he preferred its visual appearance and the more comfortable socket. The trend of decreased motion duration toward the end of the study could indicate an increased confidence, i.e., a more precise control of the velocity leading to a faster execution of the task. It would be interesting to record also the number motions per hour for CC and compare them with these of LR. However, this was technically not possible in this study and could be subject of future investigations.

It is not possible to conclude that the functional improvements of the regression-based control between the two assessments were only due to user learning (Hahne et al., 2017), as parameters were changed during the home phase of the study. However, we believe that progressive learning was indeed the main reason for improved performance, as the increase in threshold values that we have made would potentially, if at all, decrease the speed of the motions.

The subjectively reported larger frequency of dropped items in daily life with CC (Figure 4D) seems to be in contradiction to improved reliability rating and the higher number of dropped pins within the functional assessment of LR in the beginning. However, the participant explained that these item drops in daily life were not related to the control, but rather to the geometry of the lateral grip of the Michelangelo hand. The use of different prostheses to compare control algorithms is a limiting factor. On the other hand, we previously compared CC and LR with the same prosthetic hand in laboratory conditions (Hahne et al., 2018), and found a higher performance for LR. In the present work we decided to use the system the participant was wearing in daily life before the study as baseline to compare with the system he is most familiar with. Additionally, the maximal speed of the Michelangelo hand (325 mm/s for open/close, 25 rpm for rotation) is larger than the VariPlus Speed hand (300 mm/s, 17 rpm). So a potential bias due to the speed of the prosthesis would be in favor of CC. In the direct comparison (Figure 4E), the participant rated the advantage of the regression-controlled research prosthesis with 8 out of 10 points. This evaluation includes a combination of different aspects of the prosthesis, such as controllability, socket comfort, esthetic appearance of the prosthesis, that may confound each other. The research socket was constructed by a professional orthopedic technician with an inner socket of soft silicone, similar as the socket of the participant’s own Michelangelo hand. However, the eight electrode modules for the research prosthesis had to be pressed against the skin with a certain pressure to ensure a good contact, which made the socket less comfortable. This is a clear shortcoming of the study. A possible way to mediate this problem in future experiments may be to apply small conductive inserts (Hanson, 2008) that directly integrate into the inner socket or prosthesis liner to improve the comfort for control approaches that require a larger number of EMG-channels.

In (Kuiken et al., 2016) a classification-based approach was evaluated in three participants before and after home-use. While with their system a higher number of functions could be controlled, it required a frequent retraining during the home phase. Comparing the functional performance, our participant performed better than all three participants of the classification system in the SHAP, Box-And-Blocks, and Clothespin-Relocation Test already before the home phase. After the home phase the performance generally improved in both studies. Our participant using regression still outperformed all three participants of the classification-study in almost all metrics, emphasizing the daily-life suitability of our system.

In conclusion, this eight-week home trial demonstrates in a case study that a simultaneous and proportional control of two DOFs based on LR is reliably applicable in daily life. After final adjustments of the socket and parameters in week two, the control was robust and the participant was highly satisfied with the system. For this participant, the regression-controlled prosthesis outperformed the conventionally controlled one, which the participant used daily before this study in all functional metrics. The regression model was trained with data recorded in less than 1 min, with no retraining of the regression model being required over the entire study. This suggests a practical feasibility and potential clinical relevance of the presented approach, although tests with further prosthetic users are required to show whether a regression is useful for a broader range of users.

## Data Availability Statement

The datasets generated for this study are available on request to the corresponding author.

## Ethics Statement

The studies involving human participants were reviewed and approved by Ethikkomission der Universitätsmedizin Göttingen. The patients/participants provided their written informed consent to participate in this study.

## Author Contributions

JH developed the controller, and the MATLAB framework, planned and executed the laboratory experiments, supervised the home phase, analyzed the data, and wrote the manuscript. MW, DF, and AS planned and supported the experiments, analyzed the data, and revised the manuscript. MK build and improved the socket and revised the manuscript.

## Conflict of Interest

MK was employed by company Ottobock SE & Co. KGaA. The remaining authors declare that the research was conducted in the absence of any commercial or financial relationships that could be construed as a potential conflict of interest.

## References

[B1] Ameri A. Kamavuako E. N. Scheme E. J. Englehart K. B. Parker P. A. (2014). Support vector regression for improved real-time, simultaneous myoelectric control. *IEEE Trans. Neural Syst. Rehabil. Eng.* 22 1198–1209. 10.1109/TNSRE.2014.2323576 24846649

[B2] Amsuess S. Goebel P. Graimann B. Farina D. (2014). Extending mode switching to multiple degrees of freedom in hand prosthesis control is not efficient. *Conf. Proc. IEEE Eng. Med. Biol. Soc.* 2014 658–661. 10.1109/EMBC.2014.6943677 25570045

[B3] Amsuss S. Paredes L. P. Rudigkeit N. Graimann B. Herrmann M. J. Farina D. (2013). Long term stability of surface EMG pattern classification for prosthetic control. *Conf. Proc. IEEE Eng. Med. Biol. Soc.* 2013 3622–3625. 10.1109/EMBC.2013.6610327 24110514

[B4] Beaulieu R. J. Masters M. R. Betthauser J. Smith R. J. Kaliki R. Thakor N. V. (2017). Multi-position training improves robustness of pattern recognition and reduces limb-position effect in prosthetic control. *J. Prosthetics Orthot.* 29 54–62. 10.1097/JPO.0000000000000121 28983183 PMC5624523

[B5] Cipriani C. Sassu R. Controzzi M. Carrozza M. C. (2011). “Influence of the weight actions of the hand prosthesis on the performance of pattern recognition based myoelectric control: preliminary study,” in *Proceedings of the Annual International Conference of the IEEE Engineering in Medicine and Biology Society*, Boston, MA. 10.1109/IEMBS.2011.6090468 22254633

[B6] Coapt-LLC (2019). *Coapt - Complete Control.* Available online at: http://coaptengineering.com (accessed 8 Apr 2020).

[B7] Englehart K. Hudgins B. (2003). A robust, real-time control scheme for multifunction myoelectric control. *Biomed. Eng. IEEE Trans.* 50 848–854. 10.1109/tbme.2003.813539 12848352

[B8] Fougner A. Scheme E. Chan A. D. C. Englehart K. Stavdahl O. Stavdahl O. (2011). Resolving the limb position effect in myoelectric pattern recognition. *IEEE Trans. Neural Syst. Rehabil. Eng.* 19 644–651. 10.1109/TNSRE.2011.2163529 21846608

[B9] Gijsberts A. Bohra R. González D. S. Werner A. Nowak M. Caputo B. (2014). Stable myoelectric control of a hand prosthesis using non-linear incremental learning. *Front. Neurorobot.* 8:8. 10.3389/fnbot.2014.00008 24616697 PMC3935121

[B10] Hahne J. M. Biessmann F. Jiang N. Rehbaum H. Farina D. Meinecke F. C. (2014). Linear and nonlinear regression techniques for simultaneous and proportional myoelectric control. *IEEE Trans. Neural Syst. Rehabil. Eng.* 22 269–279. 10.1109/TNSRE.2014.2305520 24608685

[B11] Hahne J. M. Graimann B. Muller K.-R. (2012). Spatial filtering for robust myoelectric control. *IEEE Trans. Biomed. Eng.* 59 1436–1443. 10.1109/TBME.2012.2188799 22374342

[B12] Hahne J. M. Markovic M. Farina D. (2017). User adaptation in myoelectric man-machine interfaces. *Sci. Rep.* 7:4437. 10.1038/s41598-017-04255-x 28667260 PMC5493618

[B13] Hahne J. M. Schweisfurth M. A. Koppe M. Farina D. (2018). Simultaneous control of multiple functions of bionic hand prostheses: Performance and robustness in end users. *Sci. Robot.* 3:eaat3630 10.1126/scirobotics.aat3630 33141685

[B14] Hanson W. J. (2008). “Conductive inserts to acquire myoelectric signals through silicone liners,” in *Proceedings of the MyoElectric Controls/Powered Prosthetics Symposium*, New Brunswick.

[B15] Hussaini A. Kyberd P. (2017). Refined clothespin relocation test and assessment of motion. *Prosthet. Orthot. Int.* 41 294–302. 10.1177/0309364616660250 27473641

[B16] Hwang H. J. Hahne J. M. Müller K. R. (2017). Real-time robustness evaluation of regression based myoelectric control against arm position change and donning/doffing. *PLoS One* 12:e0186318. 10.1371/journal.pone.0186318 29095846 PMC5667774

[B17] Jiang N. Dosen S. Muller K. R. Farina D. (2012). “Myoelectric control of artificial limbsis there a need to change focus?,” in *Proceedings of the IEEE Signal Processing Magazine*, Piscataway, NJ, 10.1109/MSP.2012.2203480

[B18] Jiang N. Englehart K. B. Parker P. A. (2009). Extracting simultaneous and proportional neural control information for multiple degree of freedom prostheses from the surface electromyographic signal. *Biomed. Eng. IEEE Trans.* 56 1070–1080. 10.1109/TBME.2008.2007967 19272889

[B19] Khushaba R. N. Al-Timemy A. Kodagoda S. Nazarpour K. (2016). Combined influence of forearm orientation and muscular contraction on EMG pattern recognition. *Expert Syst. Appl.* 61 154–161. 10.1016/J.ESWA.2016.05.031

[B20] Kuiken T. Miller L. Turner K. Hargrove L. (2016). A comparison of pattern recognition control and direct control of a multiple degree-of-freedom transradial prosthesis. *IEEE J. Transl. Eng. Heal. Med.* 4 1–8. 10.1109/JTEHM.2016.2616123 28560117 PMC5396910

[B21] Kyberd P. J. Murgia A. Gasson M. Tjerks T. Metcalf C. Chappell P. H. (2009). Case studies to demonstrate the range of applications of the southampton hand assessment procedure. *Br. J. Occup. Ther.* 72 212–218. 10.1177/030802260907200506

[B22] Light C. M. Chappell P. H. Kyberd P. J. (2002). Establishing a standardized clinical assessment tool of pathologic and prosthetic hand function: normative data, reliability, and validity. *Arch. Phys. Med. Rehabil.* 83 776–783. 10.1053/apmr.2002.32737 12048655

[B23] Mathiowetz V. Volland G. Kashman N. Weber K. (1985). Adult norms for the box and block test of manual dexterity. *Am. J. Occup. Ther.* 39 386–391. 10.5014/ajot.39.6.386 3160243

[B24] Muzumdar A. (2004). *Powered Upper Limb Prostheses: Control, Implementation And Clinical Application.* Berlin: Springer.

[B25] Oskoei M. A. Hu H. (2007). Myoelectric control systems—a survey. *Biomed. Signal Process. Control* 2 275–294. 10.1016/j.bspc.2007.07.009

[B26] Ottobock (2019). *Ottobock.* Available online at: https://www.ottobock.com/en/press/press-releases/news-detail-page_24962.html (accessed February 28, 2019).

[B27] Peerdeman B. Boere D. Witteveen H. Hermens H. Stramigioli S. (2011). Myoelectric forearm prostheses: state of the art from a user-centered perspective. *J. Rehabil. Res. Dev.* 48:719. 10.1682/JRRD.2010.08.0161 21938658

[B28] Scheme E. Englehart K. (2011). Electromyogram pattern recognition for control of powered upper-limb prostheses: state of the art and challenges for clinical use. *J. Rehabil. Res. Dev.* 48 643–659. 21938652 10.1682/jrrd.2010.09.0177

[B29] Vidovic M. M. C. Hwang H. J. Amsuss S. Hahne J. M. Farina D. Muller K. R. (2016). Improving the robustness of myoelectric pattern recognition for upper limb prostheses by covariate shift adaptation. *IEEE Trans. Neural Syst. Rehabil. Eng.* 24 961–970. 10.1109/TNSRE.2015.2492619 26513794

[B30] Young A. J. Hargrove L. J. Kuiken T. A. (2011). The effects of electrode size and orientation on the sensitivity of myoelectric pattern recognition systems to electrode shift. *IEEE Trans. Biomed. Eng.* 58 2537–2544. 10.1109/TBME.2011.2159216 21659017 PMC4234036

