# Supplementary figures and images for: Longitudinal Case Study of Regression-Based Hand Prosthesis Control in Daily Life
Source: Front Neurosci. 2020 Jun 17;14:600. doi: 10.3389/fnins.2020.00600 (PMC7318897; doi:10.3389/fnins.2020.00600)

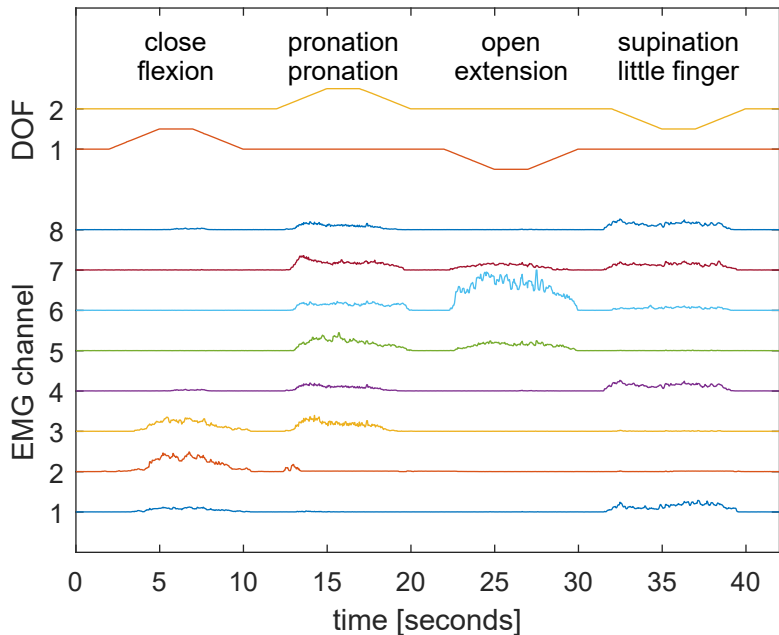

Figure S1: Representation of the eight EMG-envelopes and data labels for the training

Supplement: Supplementary file 1 [file Image_1.pdf]
