# Supplementary material for: Longitudinal Case Study of Regression-Based Hand Prosthesis Control in Daily Life
Source: Front Neurosci. 2020 Jun 17;14:600. doi: 10.3389/fnins.2020.00600 (PMC7318897; doi:10.3389/fnins.2020.00600)
Supplement: Supplementary file 2 [file Table_1.pdf]

Questionnaire

Q1	*How reliable do you perceive the control of your research/own prosthesis? (0: not at all, 10: very reliable)*
Q2	*As how natural did you perceive the control with your research/own prosthesis? (0: not at all, 10: very natural)*
Q3	*To which extent did you perceive your research/own prosthesis as your own hand? (0: not at all, 10: very much)*
Q4	*How often does it happen that you drop items or they are released unintendedly with your research/own prosthesis? (0: not at all, 10: very often)*
Q5	*How would you rate the usability of the research prosthesis compared to your own prosthesis (0: very low, 5: equal, 10: very high)?*

Table S1: Questions the participant was asked to answer for the first and the last week of the study to grade different aspect of the regression based research prostheses and his how prosthesis.
